# Hospitals and Spirit Tax

**Published:** 1915-07-03

**Authors:** F. J. Frankau

**Affiliations:** St. George's Hospital.


### THE EDITOR'S LETTER-BOX.

### Hospitals and Spirit Tax.

*To the Editor of* The Hospital.

Sir,—The article in your last issue on the above important subject, when dealing with the definition of "public hospital" in the amendment of the Finance Bill, suggests that some critics may think that "partly supported by public or charitable funds or by voluntary subscriptions" will open the door to bogus institutions. I do not pretend to judge if this is well founded, but I submit that the wording does not necessarily bear the construction you put upon it—viz., as if the word "partly" governed not only "out of public or charitable funds," but "by voluntary subscriptions." I should like to feel sure that it did so, since most hospitals are supported "partly out of . . . charitable funds" (*i.e.* endowments, legacies, etc.) *and* "partly by voluntary subscriptions," and hardly any by either of these apart from the other.

The Charitable Trusts Act, when speaking of what the judges have called "mixed charities" (for the purpose of exemption from the jurisdiction of the Commissioners) cover the case of a hospital which exists "partly from endowments *and*" (not "or") "partly by voluntary subscriptions"; and I think the amendment should be amended in this sense. In any case it should not be left to the Commissioners of Customs and Excise or any other executive body "to discriminate."—I am, etc., F. J. Frankau.

St. George's Hospital.
June 26, 1915.

[The debate on this clause in the House of Commons on Tuesday night, and Mr. Frankau's point, are considered in a leading article.—Ed. The Hospital.]

